# An Adversarial Deep-Learning-Based Model for Cervical Cancer CTV Segmentation With Multicenter Blinded Randomized Controlled Validation

**DOI:** 10.3389/fonc.2021.702270

**Published:** 2021-08-19

**Authors:** Zhikai Liu, Wanqi Chen, Hui Guan, Hongnan Zhen, Jing Shen, Xia Liu, An Liu, Richard Li, Jianhao Geng, Jing You, Weihu Wang, Zhouyu Li, Yongfeng Zhang, Yuanyuan Chen, Junjie Du, Qi Chen, Yu Chen, Shaobin Wang, Fuquan Zhang, Jie Qiu

**Affiliations:** ^1^Department of Radiation Oncology, Peking Union Medical College Hospital, Chinese Academy of Medical Sciences and Peking Union Medical College, Beijing, China; ^2^Department of Nuclear Medicine, Sun Yat-Sen University Cancer Center, Guangzhou, China; ^3^Department of Radiation Oncology, City of Hope National Medical Center, Duarte, CA, United States; ^4^Key Laboratory of Carcinogenesis and Translational Research (Ministry of Education/Beijing), Department of Radiation Oncology, Peking University Cancer Hospital and Institute, Beijing, China; ^5^Department of Radiation Oncology, Affiliated Cancer Hospital & Institute of Guangzhou Medical University, Guangzhou, China; ^6^Department of Radiation Oncology, The Fourth Hospital of Jilin University (FAW General Hospital), Jilin, China; ^7^Oncology Department, Cangzhou Hospital of Integrated Traditional Chinese and Western Medicine, Hebei, China; ^8^Department of Radiation Oncology, Yangquan First People’s Hospital, Shanxi, China; ^9^Research and Development Department, MedMind Technology Co., Ltd., Beijing, China

**Keywords:** deep-learning, auto-segmentation, evaluation, cervical cancer, radiotherapy, clinical target volume

## Abstract

**Purpose:**

To propose a novel deep-learning-based auto-segmentation model for CTV delineation in cervical cancer and to evaluate whether it can perform comparably well to manual delineation by a three-stage multicenter evaluation framework.

**Methods:**

An adversarial deep-learning-based auto-segmentation model was trained and configured for cervical cancer CTV contouring using CT data from 237 patients. Then CT scans of additional 20 consecutive patients with locally advanced cervical cancer were collected to perform a three-stage multicenter randomized controlled evaluation involving nine oncologists from six medical centers. This evaluation system is a combination of objective performance metrics, radiation oncologist assessment, and finally the head-to-head Turing imitation test. Accuracy and effectiveness were evaluated step by step. The intra-observer consistency of each oncologist was also tested.

**Results:**

In stage-1 evaluation, the mean DSC and the 95HD value of the proposed model were 0.88 and 3.46 mm, respectively. In stage-2, the oncologist grading evaluation showed the majority of AI contours were comparable to the GT contours. The average CTV scores for AI and GT were 2.68 *vs.* 2.71 in week 0 (*P* = .206), and 2.62 *vs.* 2.63 in week 2 (*P* = .552), with no significant statistical differences. In stage-3, the Turing imitation test showed that the percentage of AI contours, which were judged to be better than GT contours by ≥5 oncologists, was 60.0% in week 0 and 42.5% in week 2. Most oncologists demonstrated good consistency between the 2 weeks (*P* > 0.05).

**Conclusions:**

The tested AI model was demonstrated to be accurate and comparable to the manual CTV segmentation in cervical cancer patients when assessed by our three-stage evaluation framework.

## Introduction

Cervical cancer (CC) remains one of the leading causes of cancer-related deaths in women worldwide (1). The majority of cervical cancer cases are diagnosed at the locally advanced stage in developing countries (2). External beam radiotherapy (EBRT) with concurrent chemotherapy followed by brachytherapy, also known as radical radiotherapy (RT), is the standard treatment for locally advanced cervical cancer (3) and has been shown to be effective in decreasing the risk of pelvic and vaginal vault recurrence (4).

Accurate and individualized clinical target volume (CTV) definition is vitally important for the definitive treatment of CC (5). During the past few years, a few high-performance deep-learning models based on convolutional neural networks (CNNs) have made tremendous progress and shown promise to serve as excellent assistance for target segmentation (6–12).

A recent study has first applied a deep-learning-based method called DpnUNet to CTV segmentation in cervical cancer. The authors’ previous experimental results demonstrated that 88.65% of the contours generated by DpnUNet were acceptable for clinical usage (13). The mean dice similarity coefficient (DSC) and the 95^th^ Hausdorff distance (95HD) were 0.86 and 5.34 for the delineated CTVs. However, there are still some glaring deficits. First, performance metrics such as mean DSC and 95HD are objective and offer good reproducibility (14–17), but do not incorporate physician’s judgment and may not effectively evaluate for accuracy and applicability in a practical clinical context. Second, although the subjective oncologists’ assessments showed that most predicted contours were acceptable for clinical usage when a head-to-head comparison was conducted between manual and AI-generated contours in the same CT slice, the DpnUNet model performed inferiorly. Therefore, it indicated that the currently proposed models did not perform exactly comparably well to manual delineations in clinical practice. Moreover, it seems that the current evaluation system for automatic segmentation models remains limited and insufficient.

Given the aforementioned reasons, a novel adversarial deep-learning-based auto-segmentation model is hence proposed for CTV delineation in cervical cancer. Then a challenging three-stage multicenter randomized controlled evaluation system is designed to directly validate the model and to minimize the inter‐ and intra-observer variability. This evaluation system is a combination of objective performance metrics, subjective radiation oncologist assessment, and finally, the Turing imitation test. Accuracy and effectiveness were evaluated step by step.

## Materials and Methods

### Network Architecture

**Figure 1:**
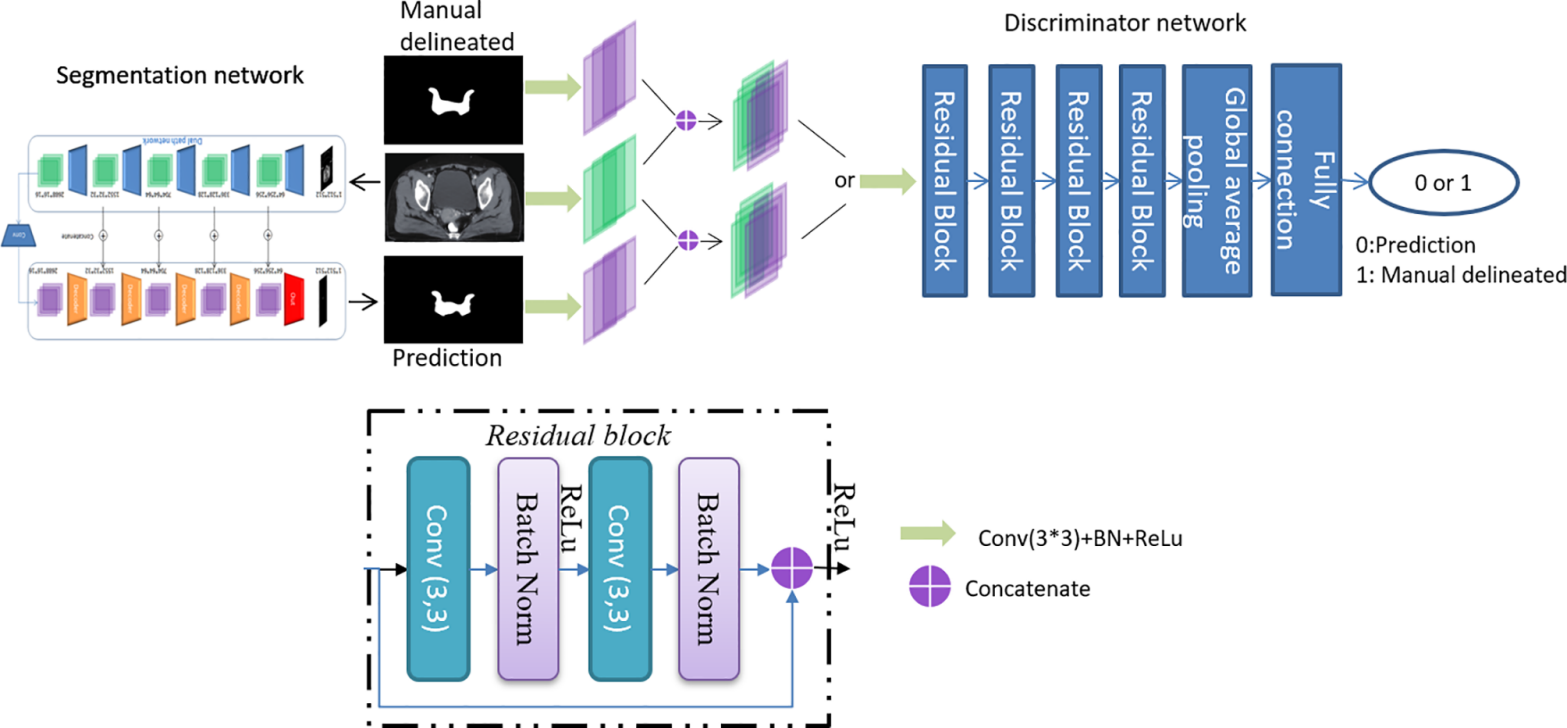
The overall architecture of the proposed model.

CTVs are challenged to be evaluated with mathematical indicators due to fuzzy boundaries and large variations among different centers and observers. Inspired by a previously described work (18), an adversarial training approach based on the typical segmentation model is proposed to achieve similar performance between CTVs delineated by the proposed model and the oncologists. The overall architecture is shown in **Figure 1**.

The proposed model is based on DpnUNet (13), which originated from the architecture of U-Net (19), but replaces all the encoder and decoder components with DPN components. Considering that the original DpnUNet is still underperforming compared with manual delineation in clinical practice, an extra convolutional layer is added at the end of DpnUNet, in which the output channels are one and the kernel size is 1 × 1. A ResNet-10 with binary classification is used as the discriminator network (20). Since the discriminator is trained to identify the input segmentation generated by the model or delineated by oncologists, it will feedback the results to the model to promote similarities between the predicted CTVs and manual delineations.

The model was trained and tested using sets of CT data from 237 patients with locally advanced cervical cancer in our center with a GTX 1080GPU. All data using oral and IV contrast were constructed with a size of 512 × 512 pixels and acquired with a Brilliance CT Big Bore (Philips Healthcare, Best, Netherlands). The proposed model was trained over 50 circles to select the best model according to the lowest validation loss score.

### Data Acquisition

To perform the three-stage evaluation, CT scans of a separate set of 20 new validation patients with locally advanced cervical cancer undergoing intensity-modulated radiation therapy (IMRT) were collected from November 2018 to December 2018 at the Peking Union Medical College Hospital. All patients were diagnosed with FIGO stage IB1–IIIC1 and/or node metastasis positive (N+) CC, treated with EBRT and radical RT. The average age ± standard deviation of these patients was 51.90 ± 12.63 years old.

CTV contours of 20 patients were redefined and re-delineated manually by radiation oncologists following the updated Radiation Therapy Oncology Group (RTOG) protocols (21–23). The CTV contours included the whole cervix, uterus, parametrium, vagina for 2 cm below GTV, and the elective nodal volume. All the contours were first reviewed by two senior radiation oncologists with more than 10 years of experience in radiotherapy specialized in cervical cancer at the Peking Union Medical College Hospital. To ensure the delineation quality of the human-generated CTV, the delineated contours were reviewed, modified, and approved collaboratively by a radiation oncologist committee consisting of eight senior oncologists at the Peking Union Medical College Hospital. The dataset of CT scans of 20 patients was used as a testing set of the proposed model to obtain artificial intelligence–generated contouring (AI) for performance assessment, of which 10 patients were randomly selected by Fisher-Yates shuffle for oncologist evaluation and the other 10 patients for the Turing-like test.

### The Three-Stage Multicenter Randomized Controlled Evaluation

#### Stage 1: Performance Metrics

**Figure 2:**
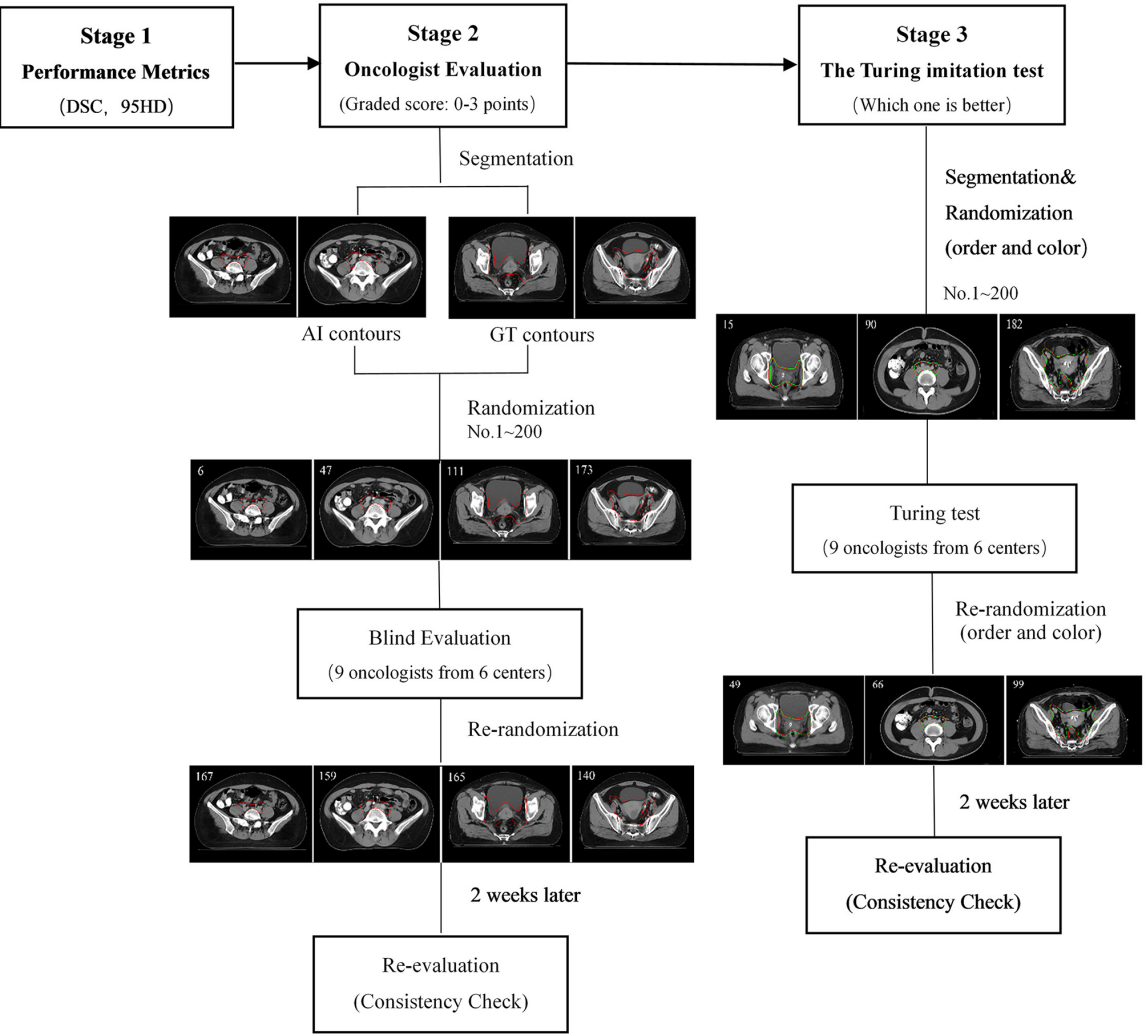
The flowchart of the three-stage multicenter randomized controlled evaluation.

The flowchart of the three-level multicenter randomized controlled evaluation is shown in **Figure 2**. During the first-stage test, the Dice similarity coefficient (DSC) and the 95th percentile Hausdorff distance (95HD) were used to quantify the performance of the proposed model objectively.

The DSC was used to measure the spatial overlap between AI and GT contours, which is defined in Eq. (1).

(1) $$\text{DSC}\;(\text{A},\;\text{B})=\frac{2|\text{A}\cap \text{B}|}{\left|\text{A}|+|\text{B}\right|}$$

Where *A* represents the volume of human-generated contour; *B* is the volume of an AI contour; and A∩B is the intersection volume that A and B have in common. The DSC value is between 0 and 1 (0 = no overlap, 1 = complete overlap).

The 95HD is defined as follows:

(2) $$95\text{HD}(\text{A},\text{B})=\text{max}\;(\text{h}(\text{A},\text{B}),\;\text{h}\;(\text{B},\text{A}),\;95^{\text{th}})$$

(3) $$\begin{array}{l} \text{HD}(\text{A},\text{B})=\text{max}(\text{h}(\text{A},\text{B}),\text{h}(\text{B},\text{A}))=\text{max}(\text{maxmin}||\text{a}-\text{b}||,\text{maxmin|}|\text{b}-\text{a}||)\\ \;\text{a}\in \text{Ab}\in \text{B b}\in \text{Ba}\in \text{A} \end{array}$$

**||•||** means the Euclidean norm of the points of A and B. The HD in mm depicts the maximum mismatch between A and B. When the HD value decreases, the overlap between A and B increases. The mean and standard deviation were calculated.

#### Stage 2: Oncologist Evaluation

Ten cases from the testing set were randomly collected for oncologist evaluation. Twenty slices from each case were randomly extracted by Fisher-Yates shuffle, of which 10 slices were randomly selected to show GT contours, and the others were overlaid with AI contours. In total, 200 slices were obtained (AI: 10 × 10 = 100 slices *vs.* GT: 10 × 10 = 100 slices) and then randomly assigned to nine experienced radiation oncologists from six different cancer centers with more than 10 years of clinical experience in cervical cancer. The dataset of 200 randomized slices was evaluated by each oncologist slice by slice. The contours were graded in four scores: 3 points (No revision), 2 points (Minor revision), 1 point (Major revision), and 0 points (Rejection). The rubric is shown in **Table 1**.

**Table 1:** Criteria for the radiation oncologist evaluation.

Score	Grade	Criteria
**3**	No revision	The segmentation is perfect and completely acceptable for treatment.
**2**	Minor revision	The segmentation needs a few minor edits but has no significant clinical impact without correction.
**1**	Major revision	The segmentation needs significant revision. Treatment planning should not proceed without contour correction.
**0**	Rejection	The segmentation is unacceptable and needs to be redrawn.

The steps are outlined as follows:

1. **Data acquisition:** Twenty slices containing CTV from 10 patients’ planning CT scans were randomly selected to generate a CT dataset consisting of 200 axial slices.
2. **Segmentation:** Both machine AI and human GT contours were generated for each dataset. Ten slices of each patient were randomly selected and overlaid with AI contours, while the other 10 slices were overlaid with GT contours. The contour color of the two groups was intentionally made the same for the blind test.
3. **Randomization:** The 200 CT slices were randomized by Fisher-Yates shuffle with an assigned unique ID so that the study authors could later distinguish whether each contour was an AI or GT.
4. **Blind evaluation:** The dataset of 200 randomized slices were distributed to the nine radiation oncologists. Each slice was scored from 0 to 3 blindly.
5. **Consistency evaluation:** After 2 weeks, the same dataset assigned in a new random order was distributed to the nine radiation oncologists for a second grading.
6. **Analysis:** The mean scores and the percentage of clinical acceptance of the AI ​​and GT groups were calculated.

#### Stage 3: The Turing Imitation Test

The Turing imitation test is a subjective head-to-head comparison between GT- and AI-generated contours. In this test, the participant was presented with two contours overlaid simultaneously in the same CT slice, one of which was generated by the AI. The radiation oncologist was requested to choose which contour was better for clinical application. The steps are outlined as follows:

1. **Data acquisition:** We randomly extracted 20 CTV containing axial CT slices from each of the remaining 10 test patients to generate a 200-slice dataset.
2. **Segmentation:** For each slice, the AI and GT contours of CTV were generated randomly in a different color (red or green). The structure colors were randomized on a per-slice basis so as not to bias the Turing imitation test.
3. **Randomization:** AI- and GT-generated CTV slices were randomized by Fisher-Yates shuffle and anonymized to facilitate the blind evaluation. Each slice was assigned a unique ID so images could be de-anonymized later to analyze.
4. **Turing test:** The dataset was distributed to the test team, consisting of nine radiation oncologists from six different centers. Each radiation oncologist was requested to compare the AI and GT delineations and select the one that was more suitable for clinical application. The evaluation time for each slice was limited to 30 s to prevent the observer from seeking additional visual clues regarding the source of the contour.
5. **Consistency evaluation:** After 2 weeks, the same dataset assigned in a new random order and color was distributed to the radiation oncologists for a new comparison.
6. **Analysis:** If the AI ​​contours received a better evaluation, the result would be considered positive. The positive rates of the entire test set and of each oncologist were calculated. Following the original Turing proposal (24), the threshold of the overall positive result rate was set to 30%. Above that, the AI model is considered to have passed the Turing imitation test.

### Statistical Analysis

The mean and standard deviation of DSC and 95HD were calculated. The Wilcoxon matched-pairs signed-rank test was used to compare the AI and GT contours in the oncologist evaluation and the Turing imitation test. The score difference between AI and GT contours evaluated by each oncologist was performed by Mann-Whitney U test. The Wilcoxon paired signed-rank test was used to compare the agreement of the oncologist evaluation between 2 weeks for each oncologist. The McNemar test was used to compare the consistency of the Turing test between 2 weeks. Statistical significance was set at two-tailed *P* <.05.

## Results

### Stage 1: Quantitative Performance Metrics

All slices of the 20 testing patients were evaluated with the quantitative performance metrics, which is shown and compared with DpnUNet in **Table 2**. The DSC and 95HD values of the proposed model were 0.88 ± 0.03 and 3.46 ± 1.88 mm, respectively.

**Table 2:** The comparison of DSC and 95HD value of our proposed model and DpnUNet.

Test	Patient (No.)	Proposed Model		DpnUNet	
		DSC	95HD (mm)	DSC	95HD (mm)
**Stage 2 patient cohort :Oncologist Evaluation**	1	0.9	1.95	0.84	2.09
	2	0.91	2.34	0.84	2.38
	3	0.9	3.68	0.89	3.61
	4	0.9	1.95	0.90	1.85
	5	0.83	7.68	0.75	8,84
	6	0.88	2.98	0.81	3.10
	7	0.84	7.07	0.80	8.10
	8	0.9	2.55	0.93	2.45
	9	0.89	2.83	0.83	3.85
	10	0.88	3.35	0.86	3.41
**Stage 3 patient cohort : The Turing Test**	11	0.85	5.1	0.75	6.17
	12	0.91	2.83	0.90	3.48
	13	0.81	7.76	0.84	7.92
	14	0.91	2.24	0.89	2.33
	15	0.91	2.21	0.94	1.97
	16	0.89	2.24	0.87	2.06
	17	0.9	2.83	0.82	2.49
	18	0.89	2.45	0.92	2.88
	19	0.93	2.25	0.94	2.26
	20	0.85	2.93	0.84	2.25
	**Mean ± STD**	0.88 ± 0.03	3.46 ± 1.88	0.86 ± 0.06	3.67 ± 2.22

### Stage 2: Oncologist Evaluation

**Figure 3:**
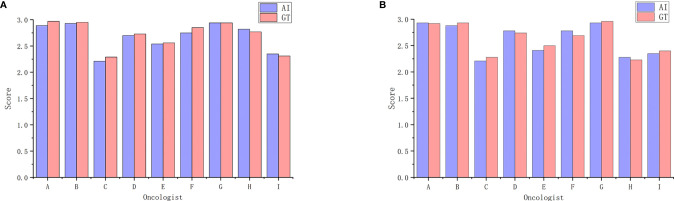
Average scores for AI and GT by the nine oncologists. **(A)** Week 0. **(B)** Week 2.

**Table 3** shows oncologist evaluation results of CTV contours. Score ≥2 was defined as suitable for clinical application. Using these scoring criteria for contour evaluation, most CTV contours were clinically acceptable by all the oncologists. For AI contours, the percentage of clinically acceptable scores was 97.4%, compared to the 98.3% of GT contours. We also compared AI and GT scores with a separate Mann-Whitney test for each oncologist and found that there was no significant difference between the week 0 timepoint and the after-2-weeks timepoint. **Figure 3** shows the CTV scores for AI and GT contours. The overall average scores for AI and GT were 2.68 *vs.* 2.71 in week 0 (*P* = .206) and 2.62 *vs.* 2.63 in week 2 (*P* = .552), respectively. The intra-observer consistency analyses between 2 weeks were performed by the Wilcoxon paired signed-rank test. It was found that the consistency of two oncologists was poor, while the others had good consistency between 2 weeks *(P* >.05).

**Table 3:** Graded oncologist evaluation for AI and GT contours.

Week 0																		
Oncologist	A		B		C		D		E		F		G		H		I	
Score	AI	GT	AI	GT	AI	GT	AI	GT	AI	GT	AI	GT	AI	GT	AI	GT	AI	GT
**3**	89%	97%	93%	95%	30%	37%	71%	74%	54%	57%	75%	85%	94%	94%	82%	77%	45%	37%
**2**	11%	3%	7%	5%	61%	56%	28%	25%	46%	42%	25%	15%	6%	6%	18%	23%	45%	57%
**1**	0	0	0	0	9%	6%	1%	1%	0	1%	0	0	0	0	0	0	10%	6%
**0**	0	0	0	0	0	1%	0	0	0	0	0	0	0	0	0	0	0	0%
**Mean Score**	2.89	2.96	2.93	2.95	2.21	2.29	2.70	2.73	2.54	2.56	2.75	2.85	2.94	2.94	2.82	2.77	2.35	2.31
**P value**	0.061		0.553		0.282		0.640		0.719		0.078		1.000		0.382		0.494	
**Week 2**																		
**3**	93%	92%	88%	93%	29%	37%	78%	77%	42%	50%	78%	69%	94%	96%	33%	33%	50%	42&
**2**	7%	8%	12%	7%	63%	54%	22%	21%	57%	50%	22%	31%	5%	4%	62%	64%	39%	56%
**1**	0	0	0	0	8%	9%	0	1%	1%	0	0	0	1%	0	5%	3%	7%	2%
**0**	0	0	0	0	0	0	0	0	0	0	0	0	0	0	0	0	4%	0
**Mean Score**	2.93	2.92	2.88	2.93	2.21	2.28	2.78	2.74	2.41	2.5	2.78	2.69	2.93	2.96	2.28	2.3	2.35	2.40
***P* value**	0.789		0.229		0.352		0.940		0.230		0.150		0.509		0.846		0.728	
**Consistency (*P* value)**		0.782		0.108		0.907		0.064		**0.007**		0.118		0.491		**0.000**		0.170

P < 0.05, the results are statistically significant.

### Stage 3: The Turing Imitation Test

**Figure 4:**
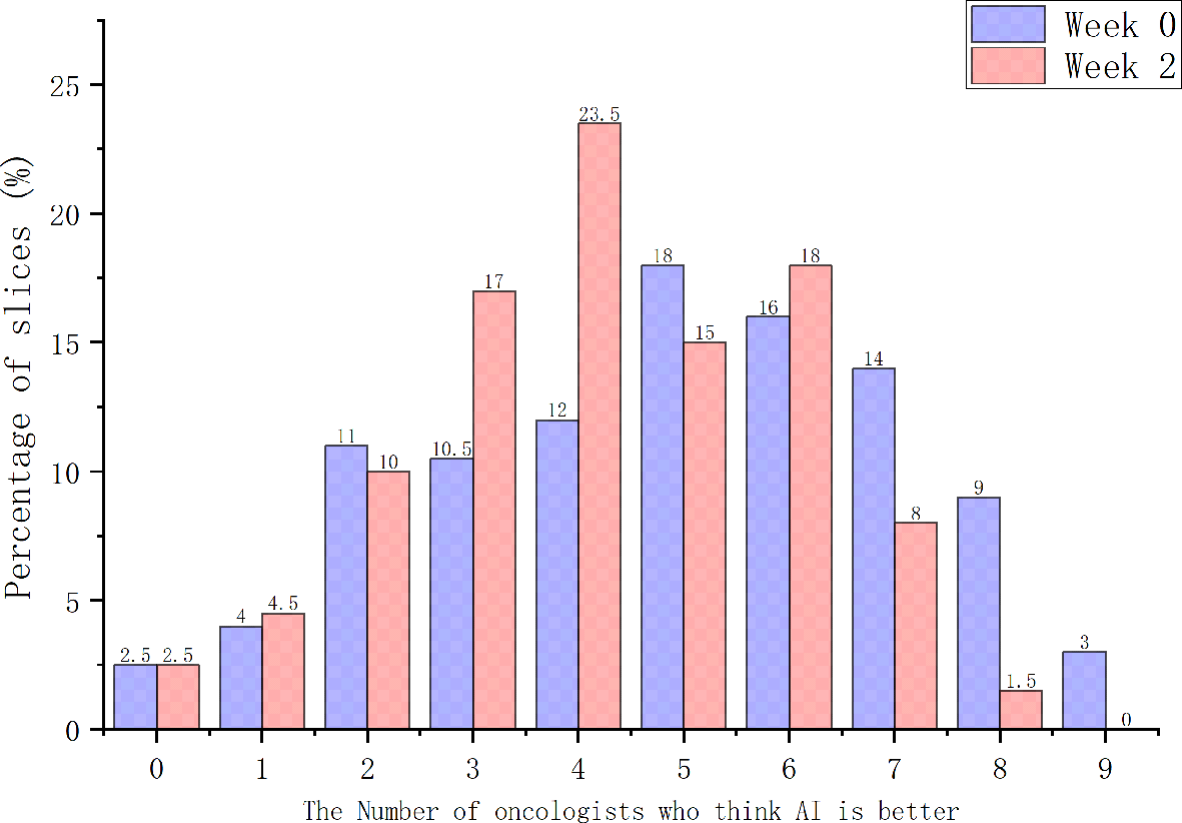
The distribution map of the positive results.

**Figure 5:**
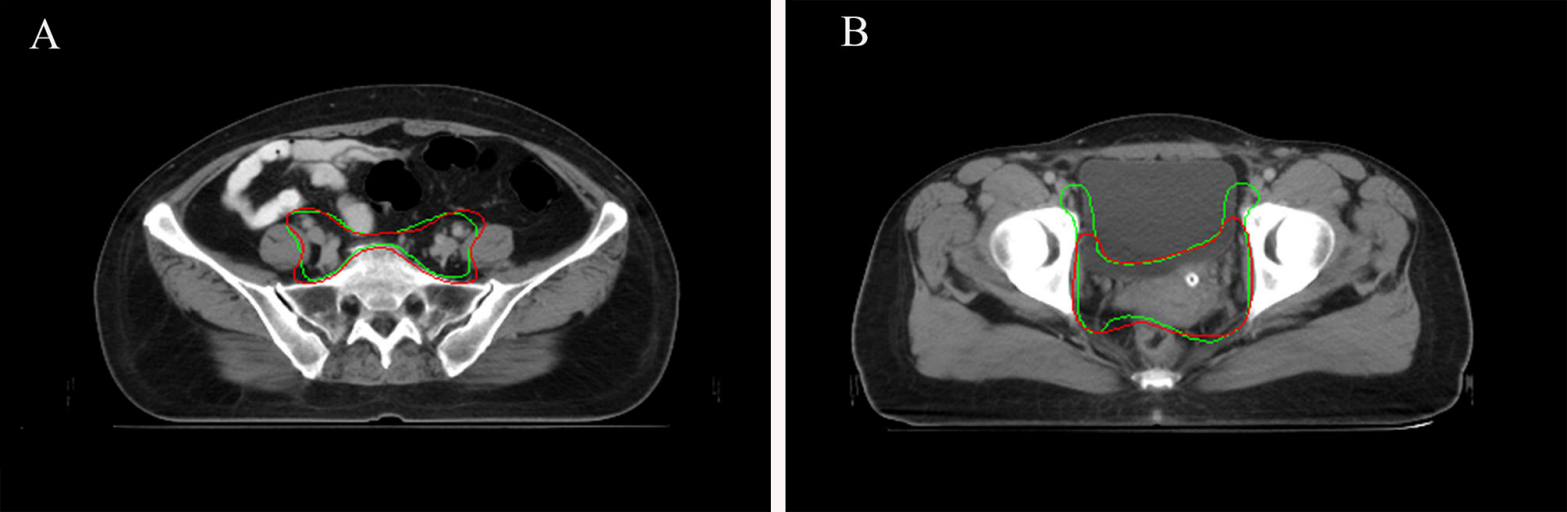
**(A)** Sample CTV where the AI contour was approved by all the oncologists. AI contours in green line. GT contours in red line. **(B)** Sample CTV where the GT contour was approved by all the oncologists. AI contours in green line. GT contours in red line.

When considering physician selection of the AI contour as preferred over the GT contour as a positive result, the overall positive rate in week 0 was 54.17% compared with 45.83% negative rate (*P* = .139), while in week 2 the positive rate was 54% *vs.* the negative rate of 46% (*P* = .128), which demonstrated the proposed deep machine learning model performed equally well or even better than human delineation. Furthermore, the consistency evaluation was performed by repeating the same dataset in different random order and colors to the test team after 2 weeks. The results are shown in **Table 4**. Subclass analysis was performed to evaluate individual oncologists and CT slices. The results showed that six slices (3.0% in week 0) of AI contours were scored to be better than GT by all the oncologists. The percentage of AI contours that were approved to be better by ≥5 oncologists was 60.0% in week 0 and 42.5% in week 2. The distribution map is shown in **Figure 4**. Sample CTV delineations are presented in **Figure 5**.

**Table 4:** The results of the Turing-like imitation test.

Oncologist	Week 0		Week 2		Consistency (*P* value)
	Positive	Negative	Positive	Negative	
A	130 (65%)	70 (35%)	137 (68.5%)	63 (31.5%)	0.296
B	92 (46%)	108 (54%)	100 (50%)	100 (50%)	0.461
C	106 (53%)	94 (47%)	116 (58%)	84 (42%)	0.134
D	107 (53.5%)	93 (46.5%)	100 (50%)	100 (50%)	0.510
E	98 (49%)	102 (51%)	114 (57%)	86 (43%)	**0.034**
F	111 (55.5%)	89 (44.5%)	102 (51%)	98 (49%)	0.508
G	122 (61%)	78 (39%)	117 (58.5%)	83 (41.5%)	0.712
H	119 (59.5%)	81 (40.5%)	95 (47.5%)	105 (52.5%)	0.101
I	90 (45%)	110 (55%)	89 (44.5%)	111(55.5%)	0.815
***P* value**	0.139		0.128		

P < 0.05, results are statistically significant.

## Discussion

Segmentation of CTV is an essential step for successful radiotherapy delivery (16). However, manual delineation is time-consuming and subjective, with considerable inter- and intra-observer variability (25–28). Therefore, accurate and consistent automated segmentation methods are highly desirable and useful for pretreatment radiotherapy planning. Automatic segmentation techniques especially based on CNN models have made significant progress with increasing reliability and accuracy in recent years, thus potentially relieving radiation oncologists from the time-cost of contouring. To the authors’ knowledge, very few studies were reported on the automatic delineation of the CTV (29–32) due to the ambiguous and blurred boundaries between the CTV and normal tissues, the potential for tumor spread or subclinical diseases in the CT images, and the inter-observer variability in recognition of anatomical structures. The current most common approach to evaluate automatic delineation of the CTV is to compare with GT contours using quantitative measures such as DSC and HD (33, 34). However, this mathematical evaluation is basic and depends only on the geometrical properties of the organ being delineated. This approach does not incorporate clinical judgment and may not adequately extract the main characteristics and the core elements of the image.

Given the clinical application, an authors’ previous study added subjective oncologist evaluation to the proposed model, and the result showed that more than 88% of the slices predicted from DpnUNet were evaluated as “No revision” or “Minor revision” (13). However, when radiation oncologists were presented with AI and GT contours overlaid simultaneously in the same CT slice, the GT contour was always the one chosen to be better. Therefore, a novel auto-segmentation model that indeed performs comparably well or even better to manual delineation for CTV delineation is desirable. Moreover, the current evaluating performance of segmentation, particularly CTV segmentation, can be challenging due to the large variations among different centers and observers (35, 36). Therefore, a three-stage randomized controlled evaluation framework was proposed, combining the three elements of traditional performance metrics, oncologist evaluation, and the Turing imitation test, for a comprehensive assessment of the proposed model in cervical cancer CTV segmentation.

During stage-1 evaluation, the mean DSC value of CTV of the proposed model was 0.88, which was higher compared with the acceptable threshold of 0.80 to 0.86 used in other studies (13, 37–39). The average 95HD value was 3.46 mm compared to 5.34 mm by the DpnUNet model (13). The results indicated a strong concordance between the proposed automatic model and human experts for CTV contouring.

In stage-2 evaluation, a multicenter randomized controlled evaluation involving nine radiation oncologists from six different centers was designed to examine the model’s clinical utility and generalization. The anonymized CT slices were randomly distributed with AI or GT contours to experienced radiation oncologists for assessment. The choice of a random design instead of using entire connected slices is mainly because AI sometimes has obvious characteristics at certain levels, especially at the beginning and the end, which do not affect the accuracy of target delineation but make it more easy to be distinguished. Moreover, the evaluation is more clinically relevant and minimizes assessment bias as oncologists are blinded to the source of the contours. The results showed that our proposed model was highly acceptable for clinical application and treatment. There was no significant difference in physician acceptability ratings between scores of AI and GT contours, which means our model can provide consistent segmentation and performed well with good agreement to the manual contours. However, there were still 2.6% of cases where the AI contours were judged by some oncologists to require major revision. We retrospectively analyzed these outlier cases and found that most of them were in the middle level of the pelvic cavity; thus, the ROIs had very unclear boundaries and massive diversity of sizes, shapes with low contrast to the rectum, bladder, and small intestines. The circumstances mentioned above limit the generalizability of the AI model, and therefore more caution is warranted.

In 1950, Alan Turing proposed an influential test for how to evaluate artificial intelligence: an imitation is successful when we cannot distinguish it from the real thing (24). Here, this analogous logic was applied to the artificial segmentation technology, and a similar Turing imitation test was proposed. The variant of the Turing imitation test used in this study is a randomized blinded evaluation. In contrast with the stage-2 task, in which evaluators viewed individual stimuli and made categorical judgments, the radiation oncologists were presented with AI and GT contour masks on the same slice and were requested to choose which was better. If the positive rate of AI is more than 30%, then the AI model was considered to have passed the test. It is a straightforward head-to-head comparison, which compares two contours in the exact same condition to minimize the interference factors such as scanning conditions, anatomical variations, and severity of disease in different patients. As shown in **Table 4**, the segmentation model passed the Turing test with overall positive rates much higher than 30%. The overall positive rate was 54.17% in week 0 and 54% in week 2, which demonstrated that the AI segmentation model performed equally well as humans (*P* = .139, *P* = .128). Moreover, correlations were observed between the objective and subjective measures. Those with lower DSC and 95HD values were also more likely to be flagged as requiring revision or inferior performance during the subjective evaluation.

Subjective assessment still has drawbacks. Oncologists involved in this study stated that they might change their opinion of the grading score if they viewed it at a later point, and may not be able to definitively decide between two contours if they showed a high degree of overlap. Therefore, the intra-observer consistency analyses between 2 weeks were performed during stage-2 and stage-3 evaluation. Most oncologists were found to maintain good consistency between 2 weeks without significant difference. Considering that good scores or positive rates could have resulted from a range of factors affecting how the contours were evaluated, a distribution map across all images involved in the Turing imitation test was additionally generated, to evaluate the number of oncologists who consistently thought AI contours were better. The results showed that the percentage of AI contours to be better than GT by ≥5 oncologists was 60.0% in week 0 and 42.5% in week 2, which further demonstrated the excellent performance of the proposed segmentation model.

## Conclusion

In this study, a novel deep-learning-based CNN model for fully automatic and accurate CTV segmentation in cervical cancer was proposed. Then a comprehensive three-stage randomized controlled evaluation framework was performed to validate the model. This evaluation system is a combination of objective and subjective evaluation and can diminish the risk of bias and enhance real-world clinical relevance compared to the most commonly used evaluation method of applying performance metrics alone. The tested AI model was demonstrated to be accurate and comparable to the manual CTV segmentation in cervical cancer patients. Furthermore, this study provided guidelines for each step, which can be referred to by other centers according to their sample size limitation. While this study focuses only on cervical cancer, the methodology and general learnings may translate to other tumor sites. Moreover, this comprehensive assessment of contouring performance may also be referenced as a base framework for evaluating the clinical utility of automatic segmentation methods in the future.

## Data Availability Statement

The original contributions presented in the study are included in the article/**Supplementary Material**. Further inquiries can be directed to the corresponding authors.

## Ethics Statement

The evaluation was reviewed and approved by the Peking Union Medical College Hospital Institutional Review board. The patients/participants provided their written informed consent to participate in this study. Written informed consent was obtained from the individual(s) for the publication of any potentially identifiable images or data included in this article.

## Author Contributions

FZ and JQ had full access to all of the data in the study and take responsibility for the integrity of the data and the accuracy of the data analysis. Concept and design: FZ, JQ, ZKL, WC. Acquisition, analysis, or interpretation of data: HG, HZ, JS, XL, JG, JY, WW, ZYL, YZ, YYC, JD. AI model design: SW, QC, YC. Drafting of the manuscript: WC, ZKL. Critical revision of the manuscript for important intellectual content: AL, RL. All authors contributed to the article and approved the submitted version.

## Funding

This work was funded by the following grants from the Non-profit Central Research Institute Fund of Chinese Academy of Medical Sciences (grant number 2019XK320014). FZ takes responsibility for the integrity of the data and the accuracy of the data analysis.

## Conflict of Interest

Authors SW, QC, and YC were employed by the company MedMind Technology Co.

The remaining authors declare that the research was conducted in the absence of any commercial or financial relationships that could be construed as a potential conflict of interest.

## Publisher’s Note

All claims expressed in this article are solely those of the authors and do not necessarily represent those of their affiliated organizations, or those of the publisher, the editors and the reviewers. Any product that may be evaluated in this article, or claim that may be made by its manufacturer, is not guaranteed or endorsed by the publisher.

## Abbreviations

CC, cervical cancer; RT, radiotherapy; EBRT, external beam radiotherapy; CTV, clinical target volume; ROIs, regions of interest; CNNs, convolutional neural networks; DPN, dual path network; GT, ground truth contouring; AI, artificial Intelligence; DICOM, digital imaging and communications in medicine; ESTRO, European Society for Radiotherapy and Oncology; RTOG, Radiation Therapy Oncology Group; DSC, Dice similarity coefficient; 95HD, the 95^th^ Hausdorff distance; IMRT, intensity modulated radiation therapy.
