# Supplementary material for: An Adversarial Deep-Learning-Based Model for Cervical Cancer CTV Segmentation With Multicenter Blinded Randomized Controlled Validation
Source: Front Oncol. 2021 Aug 19;11:702270. doi: 10.3389/fonc.2021.702270 (PMC8417437; doi:10.3389/fonc.2021.702270)
Supplement: Supplementary file 1 [file Table_1.docx]

**Suppl. Table.**

The comparison of DSC and 95HD value of all slices and random selected 20 slices from each case.

| **Test** | **Patient (No.)** | **All slices 3D** | | | **All slices 2D** | | | | | **20 slices 2D** | |
| --- | --- | --- | --- | --- | --- | --- | --- | --- | --- | --- | --- |
|  |  | **DSC** | **95HD（mm）** | **DSC** | | | **95HD（mm）** | | | **DSC** | **95HD（mm）** |
| **Stage 2**  **patient cohort：**  **Oncologist Evaluation** | 1 | 0.9 | 1.95 | | | 0.92 | | 5.14 | 0.93 | | 3.79 |
|  | 2 | 0.91 | 2.34 | | | 0.92 | | 6.14 | 0.92 | | 5.74 |
|  | 3 | 0.9 | 3.68 | | | 0.90 | | 7.23 | 0.90 | | 7.06 |
|  | 4 | 0.9 | 1.95 | | | 0.91 | | 6.38 | 0.91 | | 6.92 |
|  | 5 | 0.83 | 7.68 | | | 0.90 | | 8.76 | 0.90 | | 8.85 |
|  | 6 | 0.88 | 2.98 | | | 0.88 | | 6.49 | 0.88 | | 5.85 |
|  | 7 | 0.84 | 7.07 | | | 0.89 | | 6.10 | 0.89 | | 5.80 |
|  | 8 | 0.9 | 2.55 | | | 0.90 | | 5.95 | 0.90 | | 5.47 |
|  | 9 | 0.89 | 2.83 | | | 0.91 | | 5.78 | 0.91 | | 5.93 |
|  | 10 | 0.88 | 3.35 | | | 0.90 | | 5.57 | 0.90 | | 6.04 |
| **Stage 3**  **patient cohort：**  **The Turing Test** | 11 | 0.85 | 5.1 | | | 0.91 | | 5.64 | 0.90 | | 6.21 |
|  | 12 | 0.91 | 2.83 | | | 0.90 | | 7.27 | 0.90 | | 6.42 |
|  | 13 | 0.81 | 7.76 | | | 0.90 | | 6.59 | 0.90 | | 6.64 |
|  | 14 | 0.91 | 2.24 | | | 0.90 | | 6.67 | 0.91 | | 6.67 |
|  | 15 | 0.91 | 2.21 | | | 0.91 | | 5.68 | 0.91 | | 6.69 |
|  | 16 | 0.89 | 2.24 | | | 0.91 | | 5.87 | 0.91 | | 5.34 |
|  | 17 | 0.9 | 2.83 | | | 0.89 | | 7.37 | 0.89 | | 7.68 |
|  | 18 | 0.89 | 2.45 | | | 0.89 | | 5.91 | 0.89 | | 5.94 |
|  | 19 | 0.93 | 2.25 | | | 0.93 | | 5.15 | 0.93 | | 6.13 |
|  | 20 | 0.85 | 2.93 | | | 0.85 | | 11.20 | 0.87 | | 9.41 |
|  | **Mean±STD** | 0.88±0.03 | 3.46±1.88 | | | 0.90±0.02 | | 6.54±1.39 | 0.90±0.02 | | 6.43±1.22 |
